# The Financial Depression at the Metropolitan Hospitals To-Day and Ten Years Ago

**Published:** 1886-12-18

**Authors:** 


### Hospital Administration.

### The Financial Depression at the Metropolitan Hospitals To-day and Ten Years Ago.

[Continued from pages 5, 41, 75, 173.—Communications for this column should be addressed, "Administrator," care of the Editor of The Hospital.]

Flashes in the Pan.

No matter how obscure the disease, how serious the condition of a patient, there are always quacks in abundance who will declare they possess an infallible specific, although they may be in total ignorance of the complaint which ravishes the sufferer. So it is with hospitals, institutions, and discussions on their management. Everybody has a theory, and all declare their remedy to be the one thing needful. The financial depression at the London hospitals, its causes and removal, have afforded matter for many writers and speakers during the last fifteen years, but, to use a homely expression, the net result is that the hospital revenues do not greatly grow or improve. Is not this partly because everybody is so anxious to run his own pet nostrum and remedy, that few, if any, writers in recent times have condescended to collect the whole of the facts. Last week it was proved (p. 173) that there was no falling off to-day in the charitable revenues of the seventy-three London hospitals which are worthy of the name, as compared with ten years ago. Further, the undoubted monetary pressure, which prevails at the present time, was shown to be due to the continuance of a state of affairs which existed to an equal, and possibly to a greater extent in 1876, than it does now. This monetary pressure is caused by a deficiency in income compared with the needful expenditure of between £50,000 and £80,000 per annum. In the face of a deficiency like this, it is mere waste of time to stop to enquire how many shillings may be saved by ecomomies in the expenditure here and there. Dr. Rentoul, an unusually well-informed writer on the subject, who has won the third prize in the Sturge Competition—proves this by covering the whole economical field, from salaries and pensions to the cost of cucaine. His reductions include the practical abolition of the out-patient departments, the prevention of all abuses of hospitals by patients, and many other rather drastic reforms. But when he has exhausted all his remedies, the best result he can find it in his heart to promise from the reductions he recommends, is a total saving of £35,851 4*s.*, or less than one half of the money absolutely required to put the London Hospitals in a satisfactory financial condition. We note the *four shillings* with satisfaction, as proving the elaborate detail and care displayed in the calculations. The truth is the London hospitals want more money, and if the public will give £40,000 a year additional permanent income, then the managers may be able to meet them by attempting to enforce economies, to the extent of the balance between that sum and the actual deficiency (£80,000) in the year 1885.

**From Generalities to Particulars.**

Passing now from generalities to particulars, it may be said at once that the London Hospitals have suffered much of late years, and especially during the year 1885, from the enormous falling-off in the receipts from legacies. Thus the London hospitals received £64,286 less from legacies in 1885 than they did in the year 1876, a falling-off of more than one hundred per cent. How is this to be accounted for? Probably on two main grounds—(1) the more active interest taken in the hospitals by the public, and the increasing sense of the importance of their efficient maintenance, has had much to do with this. Formerly, the active management of most of the hospitals was confined to one or two individuals, say to half a dozen at the outside, whose fathers or relatives had preceded them in office, as the exhibition of their names and contributions on boards, or of their portraits, suspended in the Committee rooms and entrance halls testify. This system no doubt had its drawbacks, but it certainly led individual governors to regard particular hospitals as almost a part of themselves, and so to leave a substantial sum to them in their will. At present, a change of system is being brought about, the committees are larger, and the active members more numerous than formerly, with the result, that "the personal property idea" is gradually disappearing, now that the many rule, where the few were formerly supreme, and large legacies from the old source are becoming fewer. The support of the hospitals is thus being thrown more and more on the many rather than upon the few. This is matter for sincere congratulation, but the change must take time to work out satisfactorily, and hence one cause of the falling-off in extraordinary income from legacies. We welcome this change, and even the financial pressure it has caused, because it must make the management of the hospitals more and more efficient, as the managers come to realise that they have less and less of permanent and extraordinary, as opposed to charitable, income. Each generation should provide for its own sick, and this change in the incidence of hospital incomes will necessitate the enforcement of this sound principle everywhere, in our day and generation.

**Who gets the Hospital Legacies.**

(2) It has been suggested that financial difficulties are at present experienced by all classes of charities alike, and that the hospitals have not a monopoly of such evils. With the view of ascertaining how other representative charities have fared during the last ten years, the following figures have been prepared with the courteous assistance of the managers of the charities named. It must be remembered that no class of charity has to meet, to anything like the same extent that hospitals have to do, the ever increasing and urgent claims for help which continually beset them. The charities which are enumerated below are representative of very nearly every form of charitable agency, and they do not seem to have suffered a loss of charitable income, but the contrary, in the ten years ended 1885. The enormous increase in the amount received in legacies during the year 1885, as compared with 1876, may account for the falling off under that head in the hospital revenues for the same year. In any case, it is not a little remarkable; and the approximation of the figures representing the loss of income in the one case and the gain in the other, cannot fail to attract attention. The seventeen Charities, the names of which are given below,* had a combined income of £244,362 in the year 1876, derived from (1) Subscriptions and Donations, (2) Church Collections, (3) Legacies, and (4) all other Voluntary Sources, compared with a revenue of £280,366 in 1885; which gives an average in ten years of £36,004, or something like 15 per cent. Taking the items of revenue in detail, it appears that they received *from Subscriptions, etc.*, £112,409 in 1876 and £116,122 in 1885; *from Church Collections*, £9,760 in 1876 and £8,072 in 1885; *from Legacies*, £55,483 in 1876 and £94,436 in 1886, an increase of £38,953, or more than 90 per cent.; and *from other Voluntary Sources*, £66,710 in 1876 and £61,736 in 1885. Practically, the only item of revenue which does not show a falling off, or which at best does much more than maintain its own, is legacies in the case of these charitable institutions. On the other hand, the hospitals had a material increase in revenue (*see p.* 173) under nearly every head except legacies, where the falling off amounted to £64,286 or to upwards of 100 per cent. during the period under consideration. These are remarkable figures. They may only bring to light an accidental result in two particular years, which is in no sense constant or reliable. But they may also point a moral and go far to prove that the public are not now remembering the hospitals in their wills as they used to formerly, but are making their bequests to other charities in preference. In any case the point is worthy of further elucidation.

---

* Clergy Orphan Corporation, 43, Lincoln's Inn Fields, W.C.; Royal Literary Fund, 7, Adelphi Terrace, Strand, W.C.; Merchant Seamen's Orphan Asylum, Snaresbrook; Governesses' Benevolent Institution, 32, Sackville Street, W.; London Orphan Asylum, Watford, office, 181, Helen's Place, E.C.; Bishop of London's Fund, 46a Pall Mall, S.W.; British Home for Incurables, [illegible]80, Clapham Road, S.W. Office, 73, Cheapside, E.C.; Reformatory and Refuge Union, 32, Charing Cross, S.W.; Strangers' Home for Asiatics, Limehouse, E.; Royal Society for the Assistance of Discharged Prisoners, 32, Charing Cross, S.W.; Charity Organisation Society, 15, Buckingham Street, Adelphi, W.C.; London City Mission, 3, Bridewell Place, New Bridge Street, E.C.; Cripples' Nursery, 15, Park Place, Regent's Park; Society for Promoting Christian Knowledge, Northumberland Avenue; Deaf and Dumb Asylum, Old Kent Road, office, 93, Cannon Street, E.C.; National Refuges for Homeless and Destitute Children, 25, Great Queen Street, Holborn; British and Foreign Bible Society, 146, Queen Victoria Street, E.C.

